# Supplementary material for: Development of a methodology for measuring the quality of statutory social workers’ complex decision-making
Source: PLoS One. 2025 Jun 20;20(6):e0325432. doi: 10.1371/journal.pone.0325432 (PMC12180715; doi:10.1371/journal.pone.0325432)
Supplement: S1 — (DOCX) [file pone.0325432.s001.docx]

# **S1. Scoping Review Search Strings**

The search string for the Ovid platform search was as follows: ((decision-making or decision making or judg?ment*) and social work*). Fields searched included: abstract or title. Publication type excluded: book; book authored book; book edited book; chapter; circular; conference abstract; conference paper; correspondence; digital media; dissertation abstract; e-book; editorial; encyclopedia; erratum; note; online report; review. These publication types were excluded because they did not meet inclusion criteria (b) or (e).

The search string for the ProQuest platform search was as follows: (abstract ("decision making" or "decision-making" or "judgement*" or "judgment*") or title ("decision making" or "decision-making" or "judgement*" or "judgment*")) and (abstract ("social work" OR "social worker" OR "social workers" OR "social works") or title ("social work" OR "social worker" OR "social workers" OR "social works")). Publication type included: article OR feature. These publication types were included because they met inclusion criteria (b) or (e). Subject exclusions included: NOT (social work education AND medical decision making AND health services AND patients AND nurses AND health care AND students AND medical personnel AND parents & parenting AND palliative care AND training AND teaching AND hospitals AND medicine AND teachers AND clinical decision making AND clinical social work AND social policy AND education AND foster care AND management AND graduate students AND parents AND college students AND teaching methods AND adoption). These subjects were excluded because they did not meet inclusion (a).
