# Supplementary material for: Development of a methodology for measuring the quality of statutory social workers’ complex decision-making
Source: PLoS One. 2025 Jun 20;20(6):e0325432. doi: 10.1371/journal.pone.0325432 (PMC12180715; doi:10.1371/journal.pone.0325432)
Supplement: S2 — (DOCX) [file pone.0325432.s002.docx]

**Supplemental Table 1: Data Characteristics and Citations**

| **First Author (See References)** | **Short Title** | **Year** | **Adults or Children?** | **Decision-Making or Information Processing?** | **Quality Measure?** | **Quantitative or Qualitative?** | **Self-Report Method?** | **Non Self-Report Method?** | **Real Cases or Hypothetical Vignettes?** | **Vignette Source?** |
| --- | --- | --- | --- | --- | --- | --- | --- | --- | --- | --- |
| Abbotts D | Social worker decision‐making in court | 2023 | Children | Both |  | Qualitative | Individual and Group Interview, Questionnaire | No |  |  |
| Backe-Hansen, E | Justifying out-of-home placement | 2003 | Children | Decision-Making |  | Qualitative | No | Review of Case Record | Real Cases |  |
| Beckett C | Making a target work | 2018 | Children | Decision-Making | Yes | Both | Individual and Group Interview | Case Extraction |  |  |
| Benbenishty R | Information search and decision-making | 2002 | Children | Both |  | Quantitative | Questionnaire | No | Hypothetical Vignettes | Real Cases |
| Berg K | Managing reports of trouble | 2024 | Both | Decision-Making |  | Qualitative | No | Analysis of Secondary Data | Real Cases |  |
| Braye S | Deciding to use the law in social work practice | 2013 | Not stated | Decision-Making |  | Qualitative | Individual Interview | No | Real Cases |  |
| Casey B | Deconstructing discourses in assessments | 2021 | Children | Decision-Making |  | Qualitative | Individual Interview | Review of Case Record |  |  |
| Collins E | Decision making and social work in Scotland | 2011 | Both | Both |  | Qualitative | Individual Interview | Observation or Recording | Real Cases |  |
| Cook L | Making sense of the initial home visit | 2017 | Children | Decision-Making |  | Qualitative | Individual Interview | No | Real Cases |  |
| Craft J | Case factor selection | 1991 | Children | Both |  | Quantitative | Questionnaire | No | Hypothetical Vignettes | Not Identified |
| Davidson-Arad B | Social workers’ decisions on removal | 2005 | Children | Information Processing |  | Quantitative | Questionnaire | No | Real Cases |  |
| Davies M | Factors used in the detection of elder financial abuse | 2011 | Adults | Information Processing |  | Qualitative | Individual Interview | Critical Incident Review | Real Cases |  |
| Doherty P | Child protection threshold talk | 2017 | Children | Decision-Making |  | Qualitative | Individual and Group Interview | Observation or Recording | Real Cases |  |
| Drury-Hudson J | Decision-making in child protection | 1999 | Children | Decision-Making |  | Qualitative | Individual Interview | No | Hypothetical Vignettes | Not Identified |
| Durowse M (In-Text Citation 53) | Financial harm in the context of adult protection | 2024 | Adults | Both |  | Qualitative | Individual and Group Interview | Q-Sort | Hypothetical Vignettes | Research Team/Experts |
| English D | An examination of relationships | 2000 | Children | Information Processing |  | Quantitative | No | Analysis of Secondary Data | Real Cases |  |
| Enosh G | Child’s religiosity, ethnic origin and gender | 2018 | Children | Information Processing |  | Quantitative | Questionnaire | No | Hypothetical Vignettes - Factorial Survey |  |
| Enosh G | Reasoning and bias | 2015 | Children | Information Processing |  | Quantitative | Questionnaire | No | Hypothetical Vignettes - Factorial Survey | Real Cases |
| Enroos R | Family relatedness | 2023 | Children | Information Processing |  | Qualitative | Individual Interview | No | Real Cases |  |
| Holland S | Discourses of decision making | 1999 | Children | Decision-Making |  | Qualitative | Individual Interview | Case Extraction, Observation or Recording | Real Cases |  |
| Fleming S  (In-Text Citation 55) | A qualitative study of adult protection procedures | 2024 | Adults | Decision-Making |  | Qualitative | Individual Interview | No | Hypothetical Vignettes | Guidance or Textbook, Experts |
| Gillingham P | How can research and theory enhance understanding | 2023 | Children | Both |  | Qualitative | No | Review of Case Record, Case Study, Analysis Secondary Data | Real Cases |  |
| Gregory M | Story-building and narrative in social workers' case-talk | 2023 | Children | Decision-Making |  | Qualitative | Individual Interview | Observation or Recording | Real Cases |  |
| Greve RA | The importance of information processing | 2024 | Children | Decision-Making |  | Qualitative | Individual Interview | No |  |  |
| Hackett S | Decision-making in social work | 2014 | Children | Both |  | Both | Individual Interview | Case Extraction | Real Cases |  |
| Hardy M | In defence of actuarialism | 2017 | Not stated | Decision-Making |  | Qualitative | Individual Interview | Analysis of Secondary Data |  |  |
| Hayes D | Child welfare as child protection | 2014 | Children | Both |  | Both | Individual Interview | Review of Case Record, Case Study | Real Cases |  |
| Helm D | Sense-making in a social work office | 2016 | Children | Decision-Making |  | Qualitative | No | Observation or Recording | Real Cases |  |
| Keddell E | Interpreting children's best interests | 2017 | Children | Decision-Making |  | Qualitative | Individual Interview | Observation or Recording | Real Cases |  |
| Keddell E | Reasoning processes in child protection decision making | 2011 | Children | Decision-Making |  | Qualitative | Individual Interview | Critical Incident Review | Real Cases |  |
| Keddell E | Weighing it up | 2016 | Children | Decision-Making |  | Qualitative | Individual Interview | Observation or Recording |  |  |
| Keddell E | Networked decisions | 2020 | Children | Information Processing |  | Qualitative | Individual and Group Interview | No | Hypothetical Vignettes | Not Identified |
| Kettle M | The tipping point | 2017 | Children | Decision-Making |  | Qualitative | Individual Interview | Critical Incident Review | Real Cases |  |
| Killick C  (In-Text Citation 56) | Judgements of social care professionals | 2012 | Adults | Information Processing |  | Quantitative | Questionnaire | No | Hypothetical Vignettes - Factorial Survey | Guidance or Textbook, Experts |
| Mesinovic L | Sweden’s front-line: an ethnographic approach | 2023 | Children | Decision-Making |  | Qualitative | Individual and Group Interview | Observation or Recording |  |  |
| Lamponen T | Social workers’ assessment of a child’s need | 2024 | Children | Both |  | Qualitative | Group Interview | No | Hypothetical Vignettes | Research Team/Experts |
| Lev S  (In-Text Citation 54) | Social workers' perceptions regarding legal intervention | 2024 | Adults | Decision-Making |  | Qualitative | Individual Interview | No | Hypothetical Vignettes | Not Identified |
| Little J | Computer learning and risk assessment | 1998 | Children | Information Processing |  | Quantitative | No | Case Extraction |  |  |
| McCafferty P | Barriers to knowledge acquisition and utilisation | Jan 2022 | Children | Decision-Making |  | Qualitative | Individual Interview | No | Hypothetical Vignettes | Not Identified |
| McDermott F | Health social workers sources of knowledge | 2017 | Adults | Decision-Making |  | Both | Individual Interview | Case Extraction | Real Cases |  |
| McDonald A | The impact of the 2005 Mental Capacity Act | 2010 | Adults | Decision-Making |  | Qualitative | Individual Interview | Case Extraction | Real Cases |  |
| Munro E | Avoidable and unavoidable mistakes | 1996 | Children | Decision-Making |  | Quantitative | No | Analysis of Secondary Data | Real Cases |  |
| Newman C | The development of professional decision-making | 2023 | Children | Decision-Making |  | Qualitative | Individual Interview | No | Real Cases |  |
| Nouman H | Between professional norms and professionalism | 2019 | Children | Information Processing |  | Quantitative | Questionnaire | No | Hypothetical Vignettes - Factorial Survey | Real Cases |
| Nyathi N | Child protection decision-making | 2018 | Children | Decision-Making |  | Qualitative | Individual Interview | Observation or Recording | Real Cases |  |
| O'Connor L | Decision making in children and families social work | 2014 | Children | Decision-Making |  | Qualitative | Individual and Group Interview | No |  |  |
| Osmo R | Children at risk | 2004 | Children | Decision-Making |  | Qualitative | Individual Interview, Questionnaire | No | Hypothetical Vignettes | Real Cases |
| Osmo R | Social workers' strategies for treatment hypothesis testing | 2002 | Not stated | Decision-Making | Yes | Both | Questionnaire | No | Hypothetical Vignettes | Real Cases |
| Poso T | Matching children and substitute homes | 2016 | Children | Decision-Making |  | Qualitative | Individual and Group Interview | No |  |  |
| Parada H | Negotiating 'professional agency' | 2007 | Children | Decision-Making |  | Qualitative | Individual Interview | No |  |  |
| Platt D | Threshold decisions | 2006 | Children | Information Processing |  | Qualitative | Individual Interview | No | Real Cases |  |
| Platt D | Social workers' decision-making initial assessment | 2005 | Children | Decision-Making |  | Qualitative | Individual Interview | No | Real Cases |  |
| Przeperski J | Social work paradigms | 2021 | Children | Information Processing |  | Quantitative | No | Q-Sort |  |  |
| Roesch-Marsh A | Professional relationships and decision making | 2018 | Children | Decision-Making |  | Qualitative | Individual Interview | Observation or Recording | Real Cases |  |
| Saltiel D | Observing front line decision making | 2015 | Children | Decision-Making |  | Qualitative | No | Observation or Recording |  |  |
| Saltiel D | Understanding complexity in families' lives | 2013 | Children | Decision-Making |  | Qualitative | Individual Interview | Observation or Recording, Case Study | Real Cases |  |
| Segatto B | The use of discretion in decision-making | 2020 | Children | Information Processing |  | Qualitative | Group Interview | No |  |  |
| Shapira M | Modeling judgments and decisions | 1993 | Children | Information Processing |  | Quantitative | Questionnaire | No | Hypothetical Vignettes | Real Cases |
| Knighting K | Practitioners as rule using analysts | 2003 | Children | Decision-Making |  | Qualitative | Individual Interview | No | Hypothetical Vignettes | Real Cases |
| Smith Y | Beyond "common sense” | 2017 | Children | Decision-Making |  | Qualitative | Individual Interview | Observation or Recording |  |  |
| Smith Y | Rethinking decision-making | 2014 | Children | Decision-Making |  | Qualitative | Individual Interview | Observation or Recording |  |  |
| Spratt T | In and out of home care decisions | 2015 | Children | Information Processing |  | Both | Questionnaire | No | Hypothetical Vignettes - Factorial Survey | Not Identified |
| Stanley T | ‘Our tariff will rise’ | 2013 | Children | Decision-Making |  | Qualitative | Individual Interview | No | Real Cases |  |
| Stokes J | Child protection decision-making | 2012 | Children | Both |  | Quantitative | Questionnaire | No | Hypothetical Vignettes - Factorial Survey | Guidance or Textbook |
| Stokes J | Does type of harm matter? | 2014 | Children | Information Processing |  | Quantitative | Questionnaire | No | Hypothetical Vignettes - Factorial Survey | Guidance or Textbook |
| Sullivan C | Perception of risk | 2008 | Children | Information Processing |  | Quantitative | Questionnaire | No | Hypothetical Vignettes | Real Cases |
| Tufford L | Decision making factors | 2019 | Children | Both |  | Quantitative | Questionnaire | No | Hypothetical Vignettes - Factorial Survey | Experts |
| Tufford L | Decision making and relationship competence | 2021 | Children | Information Processing |  | Both | No | Simulation | Hypothetical Vignettes | Not Identified |
| Villumsen A | Informal pathways | 2023 | Children | Decision-Making |  | Qualitative | Individual Interview | No | Real Cases |  |
| Waterhouse L | Assessing child protection risk | 1992 | Children | Information Processing |  | Both | Individual Interview | No | Real Cases |  |
| Whittaker A | How do child protection practitioners make decisions | 2018 | Children | Decision-Making |  | Qualitative | Individual Interview | Observation or Recording |  |  |
| Wilkins D | Can child protection social workers forecast | 2022 | Children | Information Processing | Yes | Both | No | Case Study | Real Cases |  |
| Wilkins D | Balancing risk and protective factors | 2015 | Children | Decision-Making |  | Qualitative | Individual Interview | No | Hypothetical Vignettes | Real Cases |
| Wilkins D | Measuring the ratio of true-positive judgements | 2024 | Children | Decision-Making | Yes | Quantitative | Questionnaire | No | Hypothetical Vignettes | Real Cases |
| Yates P | Siblings as better together | 2020 | Children | Decision-Making |  | Qualitative | Individual Interview | No | Real Cases |  |
| Yates P | “It’s just the abuse that needs to stop” | 2018 | Children | Decision-Making |  | Qualitative | Individual Interview | No | Real Cases |  |
