# Supplementary material for: Development of a methodology for measuring the quality of statutory social workers’ complex decision-making
Source: PLoS One. 2025 Jun 20;20(6):e0325432. doi: 10.1371/journal.pone.0325432 (PMC12180715; doi:10.1371/journal.pone.0325432)
Supplement: S3 — (DOCX) [file pone.0325432.s003.docx]

**Supplemental Table 2: Additional Data on Findings**

| **First Author (See References)** | **Short Title** | **Publication Date** | **Sensemaking. Framing, Pattern Matching** | **Knowledge, Experience, Evidence or Heuristics, Intuition, Emotion?** | **Decision-Making Stage** | **Ethical Dilemmas: Autonomy versus Protection** | **Reference to Law** |
| --- | --- | --- | --- | --- | --- | --- | --- |
| Abbotts D | Social worker decision‐making in court | 2023 |  |  | Information Synthesis | Yes | Decision Principles, Reference to Law |
| Backe-Hansen, E | Justifying out-of-home placement | 2003 |  |  | Information Processing, Information Synthesis |  | Reference to Law |
| Beckett C | Making a target work | 2018 |  |  |  |  |  |
| Benbenishty R | Information search and decision-making | 2002 |  |  | Information Processing, Information Identification, Information Search |  |  |
| Berg K | Managing reports of trouble | 2024 | Sensemaking. Framing, Pattern Matching | Heuristics, Intuition, Emotion |  | Yes | Decision Principles, Reference to Law |
| Braye S | Deciding to use the law in social work practice | 2013 |  | Knowledge, Experience, Evidence | Information Synthesis | Yes | Legal Literacy, Decision Principles, Reference to Law |
| Casey B | Deconstructing discourses in assessments | 2021 |  |  | Information Categorising |  |  |
| Collins E | Decision making and social work in Scotland | 2011 |  | Both | Information Processing, Information Identification | Yes | Reference to Law |
| Cook L | Making sense of the initial home visit | 2017 |  | Heuristics, Intuition, Emotion | Information Synthesis |  |  |
| Craft J | Case factor selection in physical child abuse investigations | 1991 |  |  | Information Processing | Yes | Reference to Law |
| Davidson-Arad B | Social workers’ decisions on removal | 2005 |  |  | Information Processing, Information Identification |  |  |
| Davies M | Factors used in the detection of elder financial abuse | 2011 |  |  | Information Processing, Information Identification |  |  |
| Doherty P | Child protection threshold talk | 2017 | Sensemaking. Framing, Pattern Matching |  |  | Yes | Decision Principles, Reference to Law |
| Drury-Hudson J | Decision-making in child protection | 1999 |  | Knowledge, Experience, Evidence | Information Synthesis | Yes | Reference to Law |
| Durowse M (In-Text Citation 53) | Financial harm in the context of adult protection | 2024 |  | Heuristics, Intuition, Emotion | Information Processing, Information Identification, Information Synthesis |  |  |
| English D | An examination of relationships | 2000 |  |  | Information Identification |  |  |
| Enosh G | Child’s religiosity, ethnic origin and gender | 2018 |  |  | Information Processing, Information Identification | Yes | Reference to Law |
| Enosh G | Reasoning and bias | 2015 |  | Heuristics, Intuition, Emotion | Information Identification |  |  |
| Enroos R | Family relatedness | 2023 |  | Heuristics, Intuition, Emotion |  | Yes | Reference to Law |
| Holland S | Discourses of decision making | 1999 |  |  | Information Synthesis |  |  |
| Fleming S  (In-Text Citation 55) | A qualitative study of adult protection procedures | 2024 |  | Knowledge, Experience, Evidence |  |  |  |
| Gillingham P | How can research and theory enhance understanding | 2023 | Sensemaking. Framing, Pattern Matching | Heuristics, Intuition, Emotion | Information Processing, Information Synthesis |  | Reference to Law |
| Gregory M | Story-building and narrative in social workers' case-talk | 2023 | Sensemaking. Framing, Pattern Matching |  | Information Synthesis |  |  |
| Greve RA | The importance of information processing | 2024 |  | Knowledge, Experience, Evidence | Information Processing, Information Search, Information Synthesis |  |  |
| Hackett S | Decision-making in social work | 2014 |  | Heuristics, Intuition, Emotion |  |  |  |
| Hardy M | In defence of actuarialism | 2017 |  | Knowledge, Experience, Evidence | Information Synthesis |  |  |
| Hayes D | Child welfare as child protection | 2014 |  |  | Information Processing, Information Categorising, Information Synthesis | Yes | Decision Principles, Reference to Law |
| Helm D | Sense-making in a social work office | 2016 | Sensemaking. Framing, Pattern Matching |  |  |  |  |
| Keddell E | Interpreting children's best interests | 2017 | Sensemaking. Framing, Pattern Matching |  |  |  |  |
| Keddell E | Reasoning processes in child protection decision making | 2011 |  |  | Information Synthesis |  |  |
| Keddell E | Weighing it up | 2016 | Sensemaking. Framing, Pattern Matching |  |  | Yes | Reference to Law |
| Keddell E | Networked decisions | 2020 | Sensemaking. Framing, Pattern Matching |  | Information Processing, Information Identification |  |  |
| Kettle M | The tipping point | 2017 |  |  | Information Synthesis | Yes | Reference to Law |
| Killick C  (In-Text Citation 56) | Judgements of social care professionals | 2012 |  |  | Information Identification | Yes |  |
| Mesinovic L | Sweden’s front-line: an ethnographic approach | 2023 |  |  | Information Processing, Information Identification |  |  |
| Lamponen T | Social workers’ assessment of a child’s need | 2024 |  |  | Information Synthesis | Yes | Decision Principles, Reference to Law, Difficulties in Use of Law |
| Lev S  (In-Text Citation 54) | Social workers' perceptions regarding legal intervention | 2024 |  |  | Information Synthesis | Yes | Reference to Law, Difficulties in Use of Law |
| Little J | Computer learning and risk assessment | 1998 |  |  | Information Identification | Yes | Decision Principles |
| McCafferty P | Barriers to knowledge acquisition and utilisation | Jan 2022 |  | Knowledge, Experience, Evidence |  |  |  |
| McDermott F | Health social workers sources of knowledge | 2017 |  | Knowledge, Experience, Evidence |  |  |  |
| McDonald A | The impact of the 2005 Mental Capacity Act | 2010 |  |  | Information Synthesis | Yes | Decision Principles, Reference to Law |
| Munro E | Avoidable and unavoidable mistakes | 1996 | Sensemaking. Framing, Pattern Matching | Heuristics, Intuition, Emotion | Information Synthesis |  |  |
| Newman C | The development of professional decision-making | 2023 | Sensemaking. Framing, Pattern Matching | Both | Information Synthesis |  |  |
| Nouman H | Between professional norms and professionalism | 2019 |  |  | Information Identification |  |  |
| Nyathi N | Child protection decision-making | 2018 |  | Heuristics, Intuition, Emotion |  |  |  |
| O'Connor L | Decision making in children and families social work | 2014 |  | Heuristics, Intuition, Emotion |  |  |  |
| Osmo R | Children at risk | 2004 |  | Knowledge, Experience, Evidence | Information Synthesis |  |  |
| Osmo R | Social workers' strategies for treatment hypothesis testing | 2002 |  |  | Information Processing, Information Search |  |  |
| Poso T | Matching children and substitute homes | 2016 |  |  | Information Processing |  |  |
| Parada H | Negotiating 'professional agency' | 2007 |  | Knowledge, Experience, Evidence | Information Processing |  |  |
| Platt D | Threshold decisions | 2006 |  |  | Information Identification |  |  |
| Platt D | Social workers' decision-making following initial assessment | 2005 |  | Heuristics, Intuition, Emotion | Information Identification |  |  |
| Przeperski J | Social work paradigms | 2021 | Sensemaking. Framing, Pattern Matching |  | Information Synthesis | Yes | Reference to Law |
| Roesch-Marsh A | Professional relationships and decision making | 2018 |  |  | Information Processing, Information Identification, Information Search |  |  |
| Saltiel D | Observing front line decision making | 2015 |  |  | Information Processing |  |  |
| Saltiel D | Understanding complexity in families' lives | 2013 | Sensemaking. Framing, Pattern Matching |  |  |  |  |
| Segatto B | The use of discretion in decision-making | 2020 |  |  | Information Identification |  | Reference to Law |
| Shapira M | Modeling judgments and decisions | 1993 |  |  | Information Processing, Information Identification |  |  |
| Knighting K | Practitioners as rule using analysts | 2003 |  | Knowledge, Experience, Evidence |  | Yes | Decision Principles, Reference to Law |
| Smith Y | Beyond "common sense” | 2017 |  | Knowledge, Experience, Evidence | Information Processing |  |  |
| Smith Y | Rethinking decision-making | 2014 |  | Knowledge, Experience, Evidence |  |  |  |
| Spratt T | In and out of home care decisions | 2015 | Sensemaking. Framing, Pattern Matching |  | Information Identification | Yes | Reference to Law |
| Stanley T | ‘Our tariff will rise’ | 2013 |  | Heuristics, Intuition, Emotion | Information Processing, Information Synthesis |  |  |
| Stokes J | Child protection decision-making | 2012 |  | Knowledge, Experience, Evidence |  |  |  |
| Stokes J | Does type of harm matter? | 2014 |  |  | Information Processing, Information Identification |  |  |
| Sullivan C | Perception of risk | 2008 |  |  |  |  |  |
| Tufford L | Decision making factors | 2019 |  |  | Information Processing, Information Identification | Yes | Decision Principles, Reference to Law |
| Tufford L | Decision making and relationship competence | 2021 |  |  |  |  |  |
| Villumsen A | Informal pathways | 2023 |  |  | Information Identification, Information Search | Yes | Decision Principles, Reference to Law |
| Waterhouse L | Assessing child protection risk | 1992 |  |  | Information Processing, Information Identification |  |  |
| Whittaker A | How do child protection practitioners make decisions | 2018 |  | Both |  |  |  |
| Wilkins D | Can child protection social workers forecast | 2022 |  |  | Information Synthesis | Yes | Decision Principles, Reference to Law |
| Wilkins D | Balancing risk and protective factors | 2015 |  |  | Information Processing, Information Synthesis |  |  |
| Wilkins D | Measuring the ratio of true-positive judgements | 2024 |  |  | Information Synthesis |  |  |
| Yates P | Siblings as better together | 2020 | Sensemaking. Framing, Pattern Matching |  |  | Yes | Decision Principles |
| Yates P | “It’s just the abuse that needs to stop” | 2018 | Sensemaking. Framing, Pattern Matching |  |  |  |  |
