# Supplementary material for: Development of a methodology for measuring the quality of statutory social workers’ complex decision-making
Source: PLoS One. 2025 Jun 20;20(6):e0325432. doi: 10.1371/journal.pone.0325432 (PMC12180715; doi:10.1371/journal.pone.0325432)
Supplement: S4 — (DOCX) [file pone.0325432.s004.docx]

**S4. Full Text Studies Reviewed: References**

1. Abbotts D, Norman A. Social worker decision‐making in court. Child and Family Social Work. 2023. 28(2), 469–480. https://doi.org/10.1111/cfs.12977
2. Backe-Hansen E. Justifying Out-of-Home Placement: A Multiple Case Study of Decision-Making in Child Welfare and Protection Services. International Journal of Child & Family Welfare. 2003. 6. 151-166.
3. Beckett C, Dickens J. Making a target work: Messages from a pilot of the 6-month time limit on care proceedings in England. Child and Family Social Work. 2018. Aug 1;23(3):390–398. <https://doi.org/10.1111/cfs.12428>
4. Benbenishty R, Sergev D, Surkis T. Information-Search and Decision-Making by Professionals and Nonprofessionals in Cases of Alleged Child-Abuse and Maltreatment. Journal of Social Service Research, 2002. 28(3), 1–18. <https://doi.org/10.1300/J079v28n03_01>
5. Berg K, Kjellberg I. Managing reports of trouble: designated officials’ responses to reports of mistreatments initiated by service users and relatives. Journal of Elder Abuse & Neglect 2024. 36:1, pages 1-24. https://doi.org/10.1080/08946566.2023.2297221
6. Braye S, Preston-Shoot M, Wigley V. Deciding to use the law in social work practice. Journal of Social Work. 2013. vol. 13, no. 1, pp. 75-95. https://doi.org/10.1177/1468017311431476
7. Casey B, Hackett S. Deconstructing Discourses in Assessments of Child Neglect. The British Journal of Social Work. 2021. 51(6), 2097–2115. https://doi.org/10.1093/bjsw/bcab044
8. Collins E, Daly E. Decision making and social work in Scotland: the role of evidence and practice wisdom. Institute for Research and Innovation in Social Services. 2011
9. Cook L. Making sense of the initial home visit: The role of intuition in child and family social workers' assessments of risk. Journal of Social Work Practice. 2017. Vol.31(4) pp. 431-444. https://doi.org/10.1080/02650533.2017.1394826
10. Craft J, Bettin C. Case factor selection in physical child abuse investigations. Journal of Social Service Research. 1991.14(3-4), 107–123. <https://doi.org/10.1300/J079v14n03_06>
11. Davidson-Arad B, Englechin-Segal D, Wozner Y, Arieli, R. Social Workers’ Decisions on Removal: Predictions from Their Initial Perceptions of the Child’s Features, Parents’ Features, and Child’s Quality of Life. Journal of Social Service Research. 2005. 31(4), 1–23. https://doi.org/10.1300/J079v31n04_01
12. Davies M, Harries P, Cairns D, Stanley D, Gilhooly M, Gilhooly K et. al. Factors used in the detection of elder financial abuse: A judgement and decision-making study of social workers and their managers. International Social Work. 2011.54(3), 404–420. <https://doi.org/10.1177/0020872810396256>
13. Doherty P. Child protection threshold talk and ambivalent case formulations in ‘borderline’ care proceedings cases. Qualitative Social Work. 2017. 16(5), 698–716. <https://doi.org/10.1177/1473325016640062>
14. Drury-Hudson J. Decision Making in Child Protection: The Use of Theoretical, Empirical and Procedural Knowledge by Novices and Experts and Implications for Fieldwork Placement, The British Journal of Social Work. 1999. 29.1 pp. 147–169
15. Durowse M, Fenton J. Financial harm in the context of adult protection: the complexity of factors influencing joint decision-making. Journal of Adult Protection. 2024 Apr 30;26(1):36–45. https://doi.org/10.1108/JAP-08-2023-0022
16. English D, Graham C. An examination of relationships between children's protective services social worker assessment of risk and independent LONGSCAN measures of risk constructs. Children and Youth Services Review. 2000. Volume 22, Issues 11–12, pp. 897-933, https://doi.org/10.1016/S0190-7409(00)00120-1.
17. Enosh G, Nouman H, Schneck, C. Reasoning and Bias: Heuristics in Safety Assessment and Placement Decisions for Children at Risk. 2015. https://doi.org/10.1093/bjsw/bct213
18. Enosh G, Bayer-Topilsky T. Child’s Religiosity, Ethnic Origin, and Gender: A Randomized Experimental Examination of Risk Assessment and Placement Decisions in Cases of Ambiguous Risk to Children From Low SES Families, Research on Social Work Practice. 2018. Volume 29, DOI 10.1177/1049731518810795
19. Enroos R, Posp T. Family relatedness: a challenge for making decisions in child welfare. Families, Relationships and Societies. 2023. 12. 10.1332/204674321X16294377606424.
20. Fleming, S. A qualitative study of adult protection procedures: threshold screening of new referrals by designated adult safeguarding practitioners. The Journal of Adult Protection. 2024. Vol. 26 No. 1, pp. 7-23. <https://doi.org/10.1108/JAP-07-2023-0021>
21. Gillingham P, Whittaker, A. How Can Research and Theory Enhance Understanding of Professional Decision-Making in Reviews of Cases of Child Death and Serious Injury? The British Journal of Social Work. 2022. Volume 53, Issue 1, January 2023, Pages 5–22, <https://doi.org/10.1093/bjsw/bcac116>
22. Gregory M. Story-building and narrative in social workers' case-talk: A model of social work sensemaking. Child & Family Social Work. 2023. 28. https://doi.org/10.1111/cfs.13014.
23. Greve RA, Persdotter B, Christiansen, Ø, Tone, J. The Importance of Information Processing in Child Protection Cases - A Study of Social Workers' Integration of Other Professionals' Knowledge. 2023. British Journal of Social Work. 54. https://doi.org/10.1093/bjsw/bcad227.
24. Hackett S, Taylor A. (2014), Decision Making in Social Work with Children and Families: The Use of Experiential and Analytical Cognitive Processes
25. Hardy M. In Defence of Actuarialism: Interrogating the Logic of Risk in Social Work Practice.  Journal of Social Work Practice. 2017. 31(4), 395–410. https://doi.org/10.1080/02650533.2017.1394828
26. Hayes D, Spratt T. Child Welfare as Child Protection Then and Now: What Social Workers Did and Continue to Do, The British Journal of Social Work. 2014. Volume 44, Issue 3, pp. 615–635, <https://doi.org/10.1093/bjsw/bcs161>
27. Helm D. Sense-making in a social work office: an ethnogra*p*hic study of safeguarding judgements. Child & Family Social Work, 2016. 21(1), 26–35. https://doi.org/10.1111/cfs.12101
28. Holland S. Discourses of decision making in child protection: conducting comprehensive assessments in Britain, International journal of social welfare, 1999. vol. 8, no. 4, pp. 277-287. https://doi.org/10.1111/1468-2397.00094.
29. Keddell E. Reasoning processes in child protection decision making: Negotiating moral minefields and risky relationships. British Journal of Social Work. 2011. Vol.41(7), pp. 1251-1270. . https://doi.org/10.1093/bjsw/bcr012.
30. Keddell E. Weighing it up: family maintenance discourses in NGO child protection decision-making in Aotearoa/New Zealand. Child & Family Social Work. 2016. 21(4), 512–520. https://doi.org/10.1111/cfs.12168
31. Keddell E. Interpreting children's best interests: Needs, attachment and decision-making. Journal of Social Work. 2017. Vol.17(3), pp. 324-342. <https://doi.org/10.1177/1468017316644694>
32. Keddell E, Hyslop I. Networked Decisions: Decision-Making Thresholds in Child Protection. The British Journal of Social Work, 2020. 50(7), 1961–1980. https://doi.org/10.1093/bjsw/bcz131
33. Kettle M. The tipping point: Fateful moments in child protection. Child & Family Social Work. 2017. Vol.22 (Suppl 4), pp. 31-39. Kettle, Martin. 22. 10.1111/cfs.12253.
34. Killick C, Taylor B. Judgements of Social Care Professionals on Elder Abuse Referrals: A Factorial Survey. The British Journal of Social Work. 2012. 42, 814-832. <https://doi.org/10.1093/bjsw/bcr109>
35. Lamponen T, Aarnio N. Social workers’ assessment of a child’s need for services as ‘craftwork’ practice. Journal of Social Work Practice. 2024. 38. 1-14. 10.1080/02650533.2024.2302603.
36. Lev S, Waksman Y, Schindler M. Social Workers' Perceptions Regarding Legal Intervention for Older Adults without Significant Cognitive Decline Who Are Abused by Their Adult Child. J Gerontology Social Work. 2024. Jul;67(5):687-704. doi: 10.1080/01634372.2024.2339986.
37. Little J, Rixon A. Computer learning and risk assessment in child protection. Child Abuse Review. 1998. Vol.7(3), pp. 165-177. DOI:10.1002/(SICI)1099-0852(199805/06
38. McCafferty P, Taylor BJ. Barriers to knowledge acquisition and utilisation in child welfare decisions: A qualitative study. Journal of Social Work. 2022. 22(1), 87–108. <https://doi.org/10.1177/1468017320978917>
39. McDermott F, Henderson A, Quayle C. Health social workers sources of knowledge for decision making in practice. Social Work in Health Care. 2017. Vol.56(9), pp. 794-808.  doi: 10.1080/00981389.2017.1340391.
40. McDonald A. The Impact of the 2005 Mental Capacity Act on Social Workers' Decision Making and Approaches to the Assessment of Risk. The British Journal of Social Work. 2010. 40(4), 1229–1246. 40. 10.1093/bjsw/bcq021.
41. Mesinovic L, Olin E, Alstam K. Sweden’s front-line: an ethnographic approach to understanding child protection decisions. Qualitative Social Work. 2023. 23. 10.1177/14733250231207287.
42. Munro E. Avoidable and unavoidable mistakes in child protection work. British Journal of Social Work. 1996. Vol.26(6), pp. 793-808. <https://doi.org/10.1093/oxfordjournals.bjsw.a011160>
43. Newman, C., & Littlechild, B. (2022). The Development of Professional Decision-Making: A Small-Scale Study Exploring the Impact of Practice Experience on Decision-Making during Home Visits. Practice, 34(5), 370–385. https://doi.org/10.1080/09503153.2022.2048644
44. Nouman H, Enosh G, Jarjoura A. Between Professional Norms and Professionalism: Risk Assessment and Decision-Making of Arab Social Workers Regarding Children at Risk. Research on Social Work Practice. 2019. 29(5), 572–583. https://doi.org/10.1177/1049731518766677
45. Nyathi N. Child protection decision-making: social workers’ perceptions. Journal of Social Work Practice. 2018. 32(2), 189–203. https://doi.org/10.1080/02650533.2018.1448768
46. O'Connor L, Leonard K. Decision Making in Children and Families Social Work: The Practitioner's Voice. British Journal of Social Work. 2013. 44. 1805-1822. 10.1093/bjsw/bct051.
47. Osmo R, Benbenishty R. Children at risk: rationales for risk assessments and interventions, Children and Youth Services Review. 2004. Volume 26, Issue 12, pp. 1155-1173, ISSN 0190-7409. <https://doi.org/10.1016/j.childyouth.2004.05.006>.
48. Osmo R, Rosen A. Social workers strategies for treatment hypothesis testing. Social Work Research. 2002. 26(1), 9–18. <https://doi.org/10.1093/swr/26.1.9>
49. Parada H, Barnoff L, Coleman B. Negotiating 'Professional Agency': Social Work and Decision-Making within the Ontario Child Welfare System. Journal of Sociology and Social Welfare. 2007. 34. 35-56. 10.15453/0191-5096.3293.
50. Platt D. Social workers' decision-making following initial assessments of children in need in the UK. International Journal of Child and Family Welfare. 2005 Dec;8 (4):177 - 190.
51. Platt D. Threshold Decisions: How Social Workers Prioritize Referrals of Child Concern. Child Abuse Review. 2006. Vol.15(1), pp. 4-18. 15. 4 - 18. 10.1002/car.929.
52. Przeperski J. Social Work Paradigms and Their Effect on Decision Making About Out-of-Home Placement. Research on Social Work Practice. 2021. 31(4), 327–336. https://doi.org/10.1177/1049731520985607
53. Poso T, Laakso R. Matching children and substitute homes: Some theoretical and empirical notions. Child & Family Social Work. 2016. Vol.21(3), pp. 307-316. 21. 10.1111/cfs.12144.
54. Roesch-Marsh A. Professional relationships and decision making in social work: Lessons from a Scottish case study of secure accommodation decision making. Qualitative Social Work. 2018. 17(3), 405-422. <https://doi.org/10.1177/1473325016680285>
55. Knighting K, Sheppard M. Practitioners as Rule Using Analysts: A Further Development of Process Knowledge in Social Work British Journal of Social Work. 2003. 33 (2) Mar, pp.157-176. 10.1093/bjsw/33.2.157.
56. Saltiel D. Understanding complexity in families' lives: The usefulness of 'family practices' as an aid to decision-making. Child & Family Social Work. 2013. 18. 10.1111/cfs.12033.
57. Saltiel D. Observing Front Line Decision Making in Child Protection. British Journal of Social Work. 2015. 46. bcv112. 10.1093/bjsw/bcv112.
58. Segatto B, Dal Ben A, Giacomin, S. The use of discretion in decision-making by social workers at child protection services in Italy. European Journal of Social Work. 2020. 23(5), 779–789. https://doi.org/10.1080/13691457.2020.1751588
59. Shapira M, Benbenishty R. Modeling judgments and decisions in cases of alleged child abuse and neglect. Social Work Research & Abstracts. 1993. 29. 14-19. 10.1093/swra/29.2.14.
60. Smith Y. Beyond “Common Sense”: The Role of Local Knowledge in Youth Residential Treatment. Social Work Research. 2017. 41. 1-13. 10.1093/swr/svx019.
61. Smith Y. Rethinking Decision Making: An Ethnographic Study of Worker Agency in Crisis Intervention. Social Service Review. 2014. 88. 407-442. 10.1086/677846.
62. Spratt T, Devaney J, Hayes D. In and out of home care decisions: The influence of confirmation bias in developing decision supportive reasoning. Child Abuse & Neglect. 2015. 49, 76–85. <https://doi.org/10.1016/j.chiabu.2015.01.015>
63. Stanley T. ‘Our tariff will rise’: Risk, probabilities and child protection. Health. 2013. 15. 10.1080/13698575.2012.753416.
64. Stokes J, Taylor J. Does Type of Harm Matter? A Factorial Survey Examining the Influence of Child Neglect on Child Protection Decision-Making, Childcare in practice: Northern Ireland journal of multi-disciplinary childcare practice 2014. 20.4: 383–398. 10.1080/13575279.2014.905456.
65. Stokes J, Schmidt G. Child Protection Decision Making: A Factorial Analysis Using Case Vignettes Social Work. 2012. Vol. 57, No. 1, pp. 83-90
66. Sullivan C, Whitehead PC, Leschied AW, Chiodo D, Hurley D. Perception of risk among child protection workers. Children and Youth Services Review. 2008. 30(7), 699–704, https://doi.org/10.1016/j.childyouth.2007.11.010
67. Tufford L, Lee B, Bogo M, Wenghofer E, Etherington C, Thieu V, Zhao R. Decision-Making and Relationship Competence When Reporting Suspected Physical Abuse and Child Neglect: An Objective Structured Clinical Evaluation. Clinical Social Work Journal. 2021. 49. 1-15. 10.1007/s10615-020-00785-6
68. Tufford L, Lee B. Decision-Making Factors in the Mandatory Reporting of Child Maltreatment. Journal of child & adolescent trauma. 2019. 12.2: 233–244
69. Villumsen A, Søbjerg L. Informal pathways as a response to limitations in formal categorization of referrals in child and family welfare. Nordic Social Work Research. 2023. 3(2), 176–187. <https://doi.org/10.1080/2156857X.2020.1795705>
70. Waterhouse L, Carnie J. Assessing Child Protection Risk. The British Journal of Social Work. 1992. Vol. 22, no. 1, pp. 47-60, Feb. https://doi.org/10.1093/oxfordjournals.bjsw.a055830
71. Whittaker A. How Do Child-Protection Practitioners Make Decisions in Real-Life Situations? Lessons from the Psychology of Decision Making. The British Journal of Social Work. 2018. 48(7), 1967–1984. https://doi.org/10.1093/bjsw/bcx145
72. Wilkins D. Balancing risk and protective factors: How do social workers and social work managers analyse referrals that may indicate children are at risk of significant harm. British Journal of Social Work. 2015. Vol.45(1), pp. 395-411. 395-411. 10.1093/bjsw/bct114
73. Wilkins D, Meindl M. Can Child Protection Social Workers Forecast Future Actions, Events and Outcomes? a Case Study of Long-term Work with Five Families. Child Care in Practice. 2022. 1-20. 10.1080/13575279.2022.2118674.
74. Wilkins D, Meindl M. Measuring the ratio of true-positive to false-positive judgements made by child and family social workers in England: A case vignette study. Child & Family Social Work. 2024. 10.1111/cfs.13086.
75. Yates P. Siblings as better together: Social worker decision making in cases involving sibling sexual behaviour. British Journal of Social Work. 2018. Vol.48(1), pp. 176-194. 10.1093/bjsw/bcx018.
76. Yates P. “It’s Just the Abuse that Needs to Stop”: Professional Framing of Sibling Relationships in a Grounded Theory Study of Social Worker Decision Making Following Sibling Sexual Behavior, Journal of Child Sexual Abuse. 2020. 29:2, 222-245, DOI: 10.1080/10538712.2019.1692399
