# Supplementary material for: Development of a methodology for measuring the quality of statutory social workers’ complex decision-making
Source: PLoS One. 2025 Jun 20;20(6):e0325432. doi: 10.1371/journal.pone.0325432 (PMC12180715; doi:10.1371/journal.pone.0325432)
Supplement: S5 — (DOCX) [file pone.0325432.s005.docx]

# **S5. 40 Principles: Statutory Social Worker Decision-Making in Adult Social Care**

| 1 | If the professional decision is based in part on applied social science, then the social worker must make the decision based on best research evidence as evaluated by them. |
| --- | --- |
| 2 | The professional decision must be in accordance with a responsible body of professional social work opinion, even if other social workers differ in opinion. |
| 3 | To be reasonable or responsible, the professional decision must be capable of withstanding logical analysis. |
| 4 | The decision must take into account that where it appears to a local authority that an adult may have needs for care and support, the authority must assess whether the adult does have needs for care and support, and if so, what those needs are. |
| 5 | The decision to provide social work must be made as part of a care and support plan or support plan. |
| 6 | The decision must have regard to the importance of beginning with the assumption that the individual is best-placed to judge the individual’s well-being. |
| 7 | The decision must have regard to the principle that the individual may not be the best judge of their own well-being. |
| 8 | The decision must have regard to the individual’s views, wishes, feelings and beliefs. |
| 9 | The decision must have regard to the principle that it may not be appropriate to follow the individual's views, wishes and feelings. |
| 10 | The decision must have regard to the importance of preventing or delaying the development of needs for care and support or needs for support. |
| 11 | The decision must have regard to the importance of reducing needs for care and support or needs for support that already exist. |
| 12 | The decision must have regard to all the individual’s circumstances and not be based only on the individual's age or appearance or any condition of the individual's or aspect of the individual's behaviour which might lead others to make unjustified assumptions about the individual's well-being. |
| 13 | The individual must participate as fully as possible in the decision. |
| 14 | The social worker must provide the individual with the information and support necessary to enable the individual to participate in the decision. |
| 15 | The decision must have regard to the importance of achieving a balance between the individual’s well-being and the well-being of others. |
| 16 | The decision must have regard to the need to protect people from abuse and neglect. |
| 17 | The decision must have regard to the need to ensure that any restriction on the individual’s rights or freedom of action resulting from the care or support provided is kept to the minimum necessary. |
| 18 | The decision must have regard to the principle that there may be a need to use the least restrictive solution where it is necessary to interfere with the individual’s rights and freedom of action. |
| 19 | In making the decision, the social worker must co-operate with the relevant partners of the local authority and with such other persons as they consider appropriate, including other parts of the council. |
| 20 | If the individual refuses a needs assessment or a service but lacks capacity to refuse, the social worker must decide whether it would be in the individual’s best interests to accept their refusal or not. |
| 21 | The decision must have regard to the principle that the individual may be unable to protect himself or herself against abuse or neglect or the risk of it as a result of their needs for care and support and, if there is cause to suspect it, to undertake enquiries to decide whether any action should be taken and, if so, what and by whom. |
| 22 | If the individual refuses a needs assessment or a service, the social worker must decide whether the individual is experiencing, or is at risk of, abuse or neglect. |
| 23 | The decision must take into account the principle that counselling or other types of social work may be needed to help the individual to make their decisions, as distinct from providing them with advice or information. |
| 24 | The decision must not result in inhuman or degrading treatment. |
| 25 | The decision must take into account the right to respect for private and family life. The Human Rights Act requires a test of proportionality. |
| 26 | The decision must take into account the fact that any apparent decision made by an individual who lacks capacity in relation to the matter is not a decision. |
| 27 | Where there is any reason to doubt that the individual has the mental capacity to make the decision for themselves, the social worker must establish whether or not this is the case and is an appropriate professional to do so. |
| 28 | The decision must take into account that any Mental Capacity Act assessment undertaken is decision specific. |
| 29 | In making the decision the social worker must consider whether investigation is needed if an individual repeatedly makes unwise decisions that put them at significant risk of harm or exploitation or makes a particular unwise decision that is obviously irrational or out of character, in order to establish if they are under undue pressure or require more information to understand the consequences. |
| 30 | The decision must be made on the balance of probabilities. |
| 31 | The decision must be based on evidence (including inferences that can properly be drawn from the evidence). |
| 32 | The decision may rely on expert opinion, which is limited to that person’s area of expertise only. |
| 33 | The decision may rely on hearsay but direct evidence is more compelling. |
| 34 | The decision must take into account factors that ought to be taken into account. |
| 35 | The decision must not take into account factors that ought not to be taken into account. |
| 36 | The decision must not be so unreasonable that no reasonable social worker would ever consider imposing it. |
| 37 | The decision may take resources into account in how to meet need, provided eligible needs are met. |
| 38 | The decision must take into account that a local authority may not meet needs for care and support by doing anything which it or another local authority is required to do under the Housing Act 1996. |
| 39 | The local authority may not legally challenge the health care decisions of the NHS. |
| 40 | A decision that the duty to the individual is discharged may be made if a person who has the mental capacity to do so manifests a persistent and unequivocal refusal to observe reasonable requirements. |
