# Supplementary material for: Development of a methodology for measuring the quality of statutory social workers’ complex decision-making
Source: PLoS One. 2025 Jun 20;20(6):e0325432. doi: 10.1371/journal.pone.0325432 (PMC12180715; doi:10.1371/journal.pone.0325432)
Supplement: S6 — (DOCX) [file pone.0325432.s006.docx]

# **S6. Case Vignette Decision-Making Principles**

| 1 | The individual must have social care needs related to their domestic day to day life |
| --- | --- |
| 2 | The individual must have social care needs related to activities outside the home |
| 3 | The individual must have social care needs related to their interpersonal relationships |
| 4 | The individual must have social care needs related to their care services |
| 5 | The individual must have social care needs related to suitability of their accommodation |
| 6 | The individual must have needs arising from a physical illness |
| 7 | The individual must have needs arising from a physical impairment |
| 8 | The individual must have needs arising from a mental illness |
| 9 | The individual must have needs arising from a mental impairment |
| 10 | The decision must have regard to the principle that the individual might not be the best judge of their own well-being |
| 11 | The decision must have regard to the principle that it might not be appropriate to follow the individual's views, wishes and feelings |
| 12 | The decision must have regard to the importance of achieving a balance between the individual’s well-being and the well-being of others |
| 13 | The decision must have regard to the need to protect people from abuse and neglect |
| 14 | The decision must have regard to the principle that there may be a need to use the least restrictive solution where it is necessary to interfere with the individual’s rights and freedom of action |
| 15 | The decision must promote personal dignity |
| 16 | The decision must promote physical, mental and emotional well-being |
| 17 | The decision must promote protection from abuse and neglect |
| 18 | The decision must promote control by the individual over day-to-day life |
| 19 | The decision must promote work, education, training or employment |
| 20 | The decision must promote social and economic well-being |
| 21 | The decision must promote domestic, family and personal relationships |
| 22 | The decision must promote accommodation. |
| 23 | The decision must promote contribution to society. |
| 24 | The decision must have regard to the individual's views, wishes and feelings. |
| 25 | The decision have regard to the possibility that the individual might be unable to protect themselves because of their needs for care. |
| 26 | The decision must have regard to the need to ensure that any restriction on the individual’s rights or freedom of action resulting from the care or support provided is kept to the minimum necessary. |
| 27 | If the individual refuses a needs assessment or a service but lacks capacity to refuse, the social worker must decide whether it would be in the individual’s best interests to accept their refusal or not. |
| 28 | If the individual refuses a needs assessment or a service, the social worker must decide whether the individual is experiencing, or is at risk of, abuse or neglect. |
| 29 | In making the decision, the social worker must co-operate with the relevant partners of the local authority and with such other persons as they consider appropriate, including other parts of the council. |
| 30 | Where there is any reason to doubt that the individual has the mental capacity to make the decision for themselves, the social worker must establish whether or not this is the case and is an appropriate professional to do so. |
| 31 | The decision must not result in inhuman or degrading treatment. |
| 32 | The decision must take into account the right to respect for private and family life. The Human Rights Act requires a test of proportionality. |
| 33 | The local authority may not legally challenge the health care decisions of the NHS. |
| 34 | The decision may rely on expert opinion, which is limited to that person’s area of expertise only. |
| 35 | The decision may rely on hearsay but direct evidence is more compelling. |
| 36 | The decision may take resources into account in how to meet need, provided eligible needs are met. |
| 37 | A decision that the duty to the individual is discharged may be made if a person who has the mental capacity to do so manifests a persistent and unequivocal refusal to observe reasonable requirements. |
