# Supplementary material for: Development of a methodology for measuring the quality of statutory social workers’ complex decision-making
Source: PLoS One. 2025 Jun 20;20(6):e0325432. doi: 10.1371/journal.pone.0325432 (PMC12180715; doi:10.1371/journal.pone.0325432)
Supplement: S7 — (DOCX) [file pone.0325432.s007.docx]

# **S7. Principle Based Case Vignettes**

**Case Vignette AG**

AG is a 78-year-old woman living alone. She has recently been diagnosed as being in the early stages of dementia. She is an owner-occupier and lives in a two bedroom flat. Her income is from benefits and her daughter is appointee.

She has recently become friendly with a 45-year-old man. He is of Eastern European background and has come to London fairly recently to find work. Her daughter believes that this relationship is very unsuitable, especially since her mother has agreed that this man may move into her flat. The man has told the daughter that he wishes to move into AG’s flat as a paying lodger. The daughter believes that this man has developed an unhealthy influence over her mother, that her mother would not have been taken in by him in the past and that their friendship will worsen her dementia by confusing her. Her son does not disapprove in the same way and has no objections to the friendship but his contact with her mother is much more limited and he does not know the man.

The daughter pays her mother’s bills and then provides her mother with a small amount of money each week for her mother to spend as she wishes. She sees her mother daily and sometimes goes out for lunch or shopping with her. She currently does her shopping and housework for her, which her mother is now unable to do. Her children also visit their grandmother often.

This daughter has said that, if the relationship with the man continues and especially if he moves in, she will continue to manage the appointeeship and pay the bills but she will not provide her mother with cash each week as she says that this would now have to come from the lodger’s rent. She will not visit her mother daily if the friendship continues and she will stop the practical help she is providing. She will also discourage her children from their contact with their grandmother.

This daughter has contacted us and an assessment of her mother’s needs has been completed and she would be eligible for care and support for those needs which are currently being met by her daughter. A mental capacity assessment concluded that she has the capacity to make decisions about friendships. She lacks the mental capacity to make decisions about financial matters beyond the small purchases which she makes.

Her daughter says that she will happily continue to meet her mother’s needs, provided we intervene to stop the friend moving in and, if possible, stop the friendship altogether.

AG says that she wants her arrangements with her family to continue as before and that she wants her daughter to accept her friendship with this man and her decision that he may now move in.

**Case Vignette BH**

BH is a 30 year old man with schizophrenia. He is well known to the mental health services and he co-operates sufficiently with his medication. He was in 24 hour supported accommodation but a previous social worker helped him to move to a council tenancy in general needs housing. He has floating support and a befriender. Since moving in, he has been looking after himself well enough and is managing to pay his rent and utility bills well enough, with support.

The neighbours have complained through a number of channels about BH’s behaviour. They say that he is noisy throughout the night and that he deposits his rubbish in the communal areas. He lives on the ground floor in a flat with large sash windows and can regularly be seen through the windows walking naked around his flat. He has been seen urinating just outside the flats and his language and manner when spoken to is loud and obscene but he has never posed a physical threat to anyone. The neighbours say they are concerned for their children.

Partly in response to complaints to the local councillors, a Mental Health Act assessment was carried out and the conclusion was that his mental disorder was not such as to warrant detention in hospital for either assessment or treatment.

The neighbours have become very angry with BH. They have petitioned the councillors to say that it is inappropriate to have tenants with such needs living in areas with a high number of children. They say that they are not simply concerned for themselves but also for BH, who they believe is living in undignified and degrading conditions and ought to be better cared for by the council. There is also concern that some of the neighbours are aggressively angry towards BH and he has been threatened with violence on more than one occasion.

Colleagues in the Housing Department are also asking that something be done. BH is in breach of his tenancy agreement because of the disturbance he is causing to the neighbours and he is at real risk of being evicted. They don’t want to evict him but, if they have to, they will. They say that when he was in supported housing, the intensity of support enabled them, together with social care, to prevent this problem from occurring but they cannot manage it through floating support. They are looking to the social worker to resolve the problem.

BH says he was not happy in supported housing, which he felt was too restrictive and he prefers the freedom of being where he is. He is firm in his resolve not to move back to supported housing despite this now having been suggested to him by colleagues in the Housing Department many times. He says he is happy where he is and will not contemplate moving to any other tenancy. He believes that the neighbours are antagonistic towards him and that the council should do something about the threats that he has received. He does not see that the conditions in which he is living are undignified nor degrading, nor that his behaviour is anti-social. He believes people should be more tolerant. A mental capacity assessment has concluded that BH has the mental capacity to make his own decisions about the effects of his behaviour on others.

**Case Vignette CI**

CI is a woman of 52 with multiple sclerosis which affects her physical mobility but, in her case, does not include cognitive dysfunction and it has no effect on her mental capacity. She was first diagnosed when she was a young woman and started having mobility problems when she was in her mid-forties. She is a wheelchair user when outside the house and, when inside, either uses a wheelchair or is able to get around the house by supporting herself on the furniture. She has recently started having falls and has been seen by the Falls Clinic where she has also been reassessed by occupational therapy and she has a care alarm. She lives with her husband of thirty years in a house which they have owned for most of that time. Their daughter lives some distance away but visits.

CI used to be much more active. She was a school dinner lady for many years. She was always at home when her husband returned home from work and she managed the family household herself entirely. Her husband depended on her to do this and the daughter says that he has often told her that men and women have different roles in life and that it is a woman’s role to manage the home. Her husband is in fulltime work as a baggage handler at Heathrow airport. He is several years older than her and is due to retire in two months.

CI purchases her social care with direct payments. She uses the direct payments to employ a personal assistant who has not reported any concerns. In addition, the daughter visits once a week to assist her mother to bathe. The daughter recently made contact with the local authority and asked to speak confidentially. You were asked to see her and also undertake a review of the care and support plan for her mother. The daughter looked tired and anxious when she came in.

The daughter said that she believes that her father has become increasingly frustrated with the inability of his wife to look after him and their home as she used to and that she believes that he has taken to hurting her or at least that there is a risk that he will do so. She says that she sees her father being constantly angry with her mother and often frighteningly so. She says that a neighbour has told her in strict confidence that the neighbour has seen her father with his hands around her mother’s throat on a couple of occasions and that her mother then seemed in real fear. She says that the neighbour has told her that she doesn’t want to get involved. The daughter has become fearful for her mother's safety but she says she cannot raise this with her father because of the nature since childhood of her relationship with him. Nor does she want her father to know that she has reported this. She says that her father never abused her or her mother when she was growing up but he was a strict father and very distant.

There has been a general discussion with the couple in the context of your review of services, however the concerns raised by the daughter have so far been kept confidential. The general discussion covered the typical difficulties which might arise in a relationship in these changing circumstances. They have been seen together and individually. You have offered the husband a carer’s assessment which he has turned down, saying that he does not see himself as a carer.

When asked, CI and her husband both deny that there is anything wrong with their relationship. They both say that all they want from the council is the direct payments. CI says this when she is with her husband and when she is seen on her own.

**Case Vignette EK**

EK is a woman of 28 years. She has an acquired brain injury from a sports accident. There is considerable cognitive impairment, which includes difficulty with abstract concepts, processing and receiving information and with verbal fluency. This is accompanied by bouts of aggression associated with a reduction in her concern for others; a loss of ability to judge the consequences of her actions and either unawareness or denial that this is a problem. The accident has not affected her physical mobility and she is aware of the accident and the effects it has had on her life.

Prior to her accident, EK was successful and ambitious. She was an associate solicitor with a high earning capability and had bought her own flat. She was very involved in competitive sports. She had no long-term partner at the time of the accident and continues to live alone in her flat. She has supportive friends and family. Her parents live a long distance away and they are concerned for her.

EK is helped with her direct payments by her parents and has regular carers in her home. She attends a day activity centre run by Headway. This is working well but the success of the arrangements relies heavily on her day support from Headway, which is a specialist service working with people with acquired brain injuries. Her activities during the day are still highly important to her. This seems to be because it approximates as closely as is possible to a useful working life and routine. She experiences her main challenges when she is at Headway.

EK made good progress when she began attending Headway, but the benefits seem to have reached a plateau and recently she has found the experience frustrating. She wants to be busy but finds tasks which she would have considered very simple before her accident very difficult and sometimes impossible now and this causes her a great deal of frustration. Initially this led to her being verbally aggressive and more recently she has also hit the staff and these occasions are becoming more frequent. Headway have raised concerns about the need to protect their staff. They believe that, without their specialist input, EK will lose the benefits she has gained although they do not believe that she is likely to make any more improvement.

EK has also taken to wandering from Headway and walking into the busy streets which has caused her parents a great deal of concern for her safety. The staff are unable to guarantee that they will be able to divert her from doing this. Her parents are also concerned that the aggressive and violent outbursts only happen at Headway as they believe that it is only serving to frustrate her and that too much is being asked of their daughter. They believe that it would be in her better interests to stop attending but they are concerned that, if she does stop, her care arrangements in her flat will not be sufficient. They believe that she will start wandering from her flat because of the restricted space there and that the carers will not be able to provide her with enough activities during the day. They are suggesting that she should now move into a care home, which could not replicate Headway but would have what they consider to be sufficiently challenging activities on site. She would require a specialist care home and there are no such placements available near enough for her to continue to attend Headway.

EK’s wishes are to remain living in her flat and to continue to attend Headway. Her flat was very important to her before the accident as was the independence she had there and she is still very happy there. She has been assessed as no longer having the mental capacity to decide for herself where she should live or what care and support she should have during the day.
