# Supplementary material for: Development of a methodology for measuring the quality of statutory social workers’ complex decision-making
Source: PLoS One. 2025 Jun 20;20(6):e0325432. doi: 10.1371/journal.pone.0325432 (PMC12180715; doi:10.1371/journal.pone.0325432)
Supplement: S8 — (DOCX) [file pone.0325432.s008.docx]

**S9. Workbook for AG and BH Case Vignettes**

| 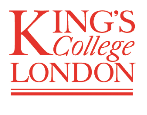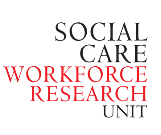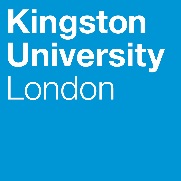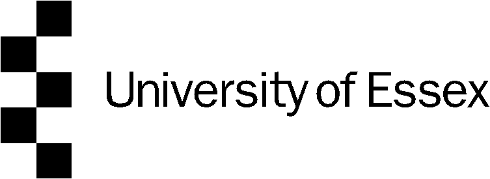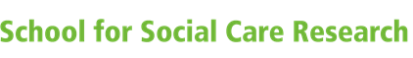 **Studying Social Work Supervision**  **This study is based on the use of case vignettes and we ask that you keep their content confidential to ensure that they can be used by others in the research without any knowledge of their content in advance.**  In this study, you are asked to review two case vignettes, each comprising a number of hypothetical facts about an imaginary social work case. You are asked to assume that this is the first information you have received on each case.  You are asked to identify the problem(s) which you as a social worker would need to address on each case. You are asked to identify no more than five.  Your main task is to identify the decision(s) or recommendation(s) you as a statutory social worker must or should take on the next steps in each case, based solely on the information available to you. You are asked to list no more than five such next steps for each case vignette.  You are also asked to identify which area of well-being as defined in the Care Act would be your highest priority to promote in each case.  You are also asked to identify which, from a list of key decision making principles, were most important in you arriving at your decisions or recommendations. This list has been given to you. They are key decision making principles which underpin statutory social work decision making. They are not exhaustive. Not all apply to all social work cases and some may be more or less relevant, depending on the facts of the case. You are asked to identify five from this list.   \| **Please enter your name:** \|  \| \| --- \| --- \|  \| **For office use only** \| \| \| --- \| --- \| \| Supervisor \| No supervisor \|   This research has been funded by the NIHR School for Social Care Research. This phase of the research has been approved by Kingston University Business Faculty Research Ethics Committee (FR 16 09) and is supported by the Association of Directors of Adult Social Services (RG15 024). The case vignettes have been co-designed with the London Principal Social Workers’ Network.  Section A  **This page is deliberately blank**  **Social Work Case Vignette AG**   1. **Please identify the problem(s) which you as a social worker would need to address on this case.**   a  b  c  d  e  **Social Work Case Vignette AG (continued)**   1. **Please list the decision(s) or recommendation(s) you as a statutory social worker must or should take on the next steps in this case.**   a  b  c  d  e  **Social Work Case Vignette AG (continued)**   1. **There is a general duty under the Care Act 2014 to promote the individual’s well-being. The well-being areas as defined in the Care Act are listed below. Please tick which ONE of these well-being areas would be your highest priority to promote in this case.**  \|  \| Personal dignity \|  \| Participation in education \| \| --- \| --- \| --- \| --- \| \|  \| Physical health \|  \| Participation in training \| \|  \| Mental health \|  \| Participation in recreation \| \|  \| Emotional well-being \|  \| Social well-being \| \|  \| Protection from abuse \|  \| Economic well-being \| \|  \| Protection from neglect \|  \| Domestic, family and personal relationships \| \|  \| Control by the individual over day-to-day life \|  \| Suitability of living accommodation \| \|  \| Participation in work \|  \| The individual’s contribution to society \|  1. **You have been given a list of Key Principles Underpinning Social Work Decision Making in the statutory sector. Please identify by Principle Number which of these have been most important for you to consider in arriving at your decision(s) or recommendation(s). You should identify five.**  \| Principle No \| Principle No \| Principle No \| Principle No \| Principle No \| \| --- \| --- \| --- \| --- \| --- \| \|  \|  \|  \|  \|  \|   **Social Work Case Vignette BH**   1. **Please identify the problem(s) which you as a social worker would need to address on this case.**   a  b  c  d  e  **Social Work Case Vignette BH (continued)**   1. **Please list the decision(s) or recommendation(s) you as a statutory social worker must or should take on the next steps in this case.**   a  b  c  d  e  **Social Work Case Vignette BH (continued)**   1. **There is a general duty under the Care Act 2014 to promote the individual’s well-being. The well-being areas as defined in the Care Act are listed below. Please tick which ONE of these well-being areas would be your highest priority to promote in this case.**  \|  \| Personal dignity \|  \| Participation in education \| \| --- \| --- \| --- \| --- \| \|  \| Physical health \|  \| Participation in training \| \|  \| Mental health \|  \| Participation in recreation \| \|  \| Emotional well-being \|  \| Social well-being \| \|  \| Protection from abuse \|  \| Economic well-being \| \|  \| Protection from neglect \|  \| Domestic, family and personal relationships \| \|  \| Control by the individual over day-to-day life \|  \| Suitability of living accommodation \| \|  \| Participation in work \|  \| The individual’s contribution to society \|  1. **You have been given a list of Key Principles Underpinning Social Work Decision Making in the statutory sector. Please identify by Principle Number which of these have been most important for you to consider in arriving at your decision(s) or recommendation(s). You should identify five.**  \| Principle No \| Principle No \| Principle No \| Principle No \| Principle No \| \| --- \| --- \| --- \| --- \| --- \| \|  \|  \|  \|  \|  \|   **Please do not turn the page until you are asked**  Section B  **We would like to know how you found completing these tasks and how you felt about them. Please indicate on the scales below how strongly you agree or disagree with each statement.** (For A-E, please find definitions below)   1. **Mental Demand:** How much mental and perceptual activity was required?  \| Low \| 1 \| 2 \| 3 \| 4 \| 5 \| 6 \| 7 \| High \| \| --- \| --- \| --- \| --- \| --- \| --- \| --- \| --- \| --- \|  1. **Temporal Demand:** How much time pressure did you feel?  \| Low \| 1 \| 2 \| 3 \| 4 \| 5 \| 6 \| 7 \| High \| \| --- \| --- \| --- \| --- \| --- \| --- \| --- \| --- \| --- \|  1. **Performance:** How successful do you think you were?  \| Poor \| 1 \| 2 \| 3 \| 4 \| 5 \| 6 \| 7 \| Good \| \| --- \| --- \| --- \| --- \| --- \| --- \| --- \| --- \| --- \|  1. **Effort:** How hard did you have to work?  \| Low \| 1 \| 2 \| 3 \| 4 \| 5 \| 6 \| 7 \| High \| \| --- \| --- \| --- \| --- \| --- \| --- \| --- \| --- \| --- \|  1. **Frustration:** How insecure did you feel during the task?  \| Low \| 1 \| 2 \| 3 \| 4 \| 5 \| 6 \| 7 \| High \| \| --- \| --- \| --- \| --- \| --- \| --- \| --- \| --- \| --- \|   **Rating Scale Definitions**  **Mental Demand:** How much mental and perceptual activity was required (e.g. thinking, deciding, calculating, remembering, looking, searching etc.)? Was the task easy or demanding, simple or complex, exacting or forgiving?  **Temporal Demand:** How much time pressure did you feel due to the rate or pace at which the tasks or task elements occurred? Was the pace slow and leisurely or rapid and frantic?  **Performance:** How successful do you think you were in accomplishing the goals of the task set by the researcher (or yourself)? How satisfied were you with your performance in accomplishing these goals?  **Effort:** How hard did you have to work (mentally) to accomplish your level of performance?  **Frustration:** How insecure, discouraged, irritated, stressed and annoyed versus secure, gratified, content, relaxed and complacent did you feel during the task?   1. To what extent would you feel able to make decisions as distinct from recommendations on these cases?  \| Completely Would Not \| 1 \| 2 \| 3 \| 4 \| 5 \| 6 \| 7 \| Completely Would \| \| --- \| --- \| --- \| --- \| --- \| --- \| --- \| --- \| --- \|  1. To what extent did your sense of authority as a social worker affect your decisions or recommendations on these cases?  \| Completely Did Not \| 1 \| 2 \| 3 \| 4 \| 5 \| 6 \| 7 \| Completely Did \| \| --- \| --- \| --- \| --- \| --- \| --- \| --- \| --- \| --- \|  1. To what extent did you feel accountable when you made your decisions or recommendations on these cases?  \| Completely Did Not \| 1 \| 2 \| 3 \| 4 \| 5 \| 6 \| 7 \| Completely Did \| \| --- \| --- \| --- \| --- \| --- \| --- \| --- \| --- \| --- \|  1. To what extent did feeling that you had to account for your decisions or recommendations on these cases affect what you decided or recommended?  \| Not At All \| 1 \| 2 \| 3 \| 4 \| 5 \| 6 \| 7 \| Completely \| \| --- \| --- \| --- \| --- \| --- \| --- \| --- \| --- \| --- \|  1. To what extent would you feel responsible for the outcome from your decisions or recommendations on these cases?  \| Not At All \| 1 \| 2 \| 3 \| 4 \| 5 \| 6 \| 7 \| Completely \| \| --- \| --- \| --- \| --- \| --- \| --- \| --- \| --- \| --- \|  1. To what extent would you feel regret if your decisions or recommendations on these cases led to a poor outcome for the client?  \| Not At All \| 1 \| 2 \| 3 \| 4 \| 5 \| 6 \| 7 \| Completely \| \| --- \| --- \| --- \| --- \| --- \| --- \| --- \| --- \| --- \|  1. How many years is it since you qualified as a social worker?   **Thank you for your participation in this research.**  **Your support is greatly appreciated.** |
| --- | --- | --- | --- | --- | --- | --- | --- | --- | --- | --- | --- | --- | --- | --- | --- | --- | --- | --- | --- | --- | --- | --- | --- | --- | --- | --- | --- | --- | --- | --- | --- | --- | --- | --- | --- | --- | --- | --- | --- | --- | --- | --- | --- | --- | --- | --- | --- | --- | --- | --- | --- | --- | --- | --- | --- | --- | --- | --- | --- | --- | --- | --- | --- | --- | --- | --- | --- | --- | --- | --- | --- | --- | --- | --- | --- | --- | --- | --- | --- | --- | --- | --- | --- | --- | --- | --- | --- | --- | --- | --- | --- | --- | --- | --- | --- | --- | --- | --- | --- | --- | --- | --- | --- | --- | --- | --- | --- | --- | --- | --- | --- | --- | --- | --- | --- | --- | --- | --- | --- | --- | --- | --- | --- | --- | --- | --- | --- | --- | --- | --- | --- | --- | --- | --- | --- | --- | --- | --- | --- | --- | --- | --- | --- | --- | --- | --- | --- | --- | --- | --- | --- | --- | --- | --- | --- | --- | --- | --- | --- | --- | --- | --- | --- | --- | --- | --- | --- | --- | --- | --- | --- | --- | --- | --- | --- | --- | --- | --- | --- | --- | --- | --- | --- | --- | --- | --- | --- | --- | --- |
