# Supplementary material for: Development of a methodology for measuring the quality of statutory social workers’ complex decision-making
Source: PLoS One. 2025 Jun 20;20(6):e0325432. doi: 10.1371/journal.pone.0325432 (PMC12180715; doi:10.1371/journal.pone.0325432)
Supplement: S9 — (DOCX) [file pone.0325432.s009.docx]

**S10. Workbook for CI and EK Case Vignettes**

| 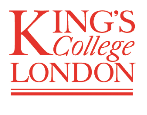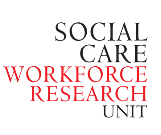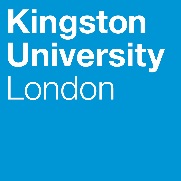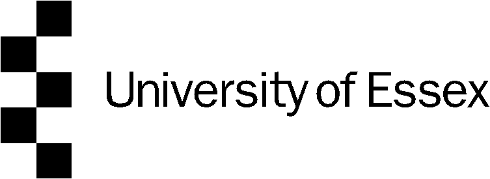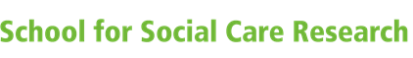 **Social Worker Supervision Research Study: Instructions**  You are asked to consider the two case studies which you have been given, each of which consists of hypothetical facts about a case suitable for a social worker. Your employer is a local authority which has asked you to undertake a s27 review of each case under their Care Act duties. Your employer has delegated to you the responsibility to make professional recommendations to your supervisor about the decisions which must or should be taken next.  **For each case study:**  **From the facts of the case, please identify up to five issues which you as a social worker would need to address, making it clear how they are problems.**  **Please recommend the decisions which must or should be taken next to address each problem identified.**  **In formulating the problems:**  **Please consider whether the service user’s perception of what is in their best interests appears to differ from your professional assessment of what is in their best interests.**  **Please also consider whether their wishes or feelings seem to present a problem in achieving what is in their best interests.**  **In formulating your recommendations:**  **If you have concluded that the service user’s perception of what is in their best interests differs from your assessment of it, please make clear the best interests outcome you are recommending.**  **If you have concluded that their wishes or feelings present a problem, please make clear the way and degree to which you recommend their wishes or feelings be taken into account.**  **Please base your responses solely on the information given in the case studies. It is assumed that you will see the adult concerned, that you will continue to collect information and that you will convene any necessary meetings. It is assumed that you have made all the appropriate links with health and housing partners and that these partners are fully and appropriately involved. Please do not include these as recommended decisions.**  Please keep the content of the case studies confidential as they will be used throughout this study  This research has been funded by the NIHR School for Social Care Research. This phase of the research has been approved by Kingston University Business Faculty Research Ethics Committee (FR 16 09) and is supported by the Association of Directors of Adult Social Services (RG15 024).  **Social Worker Supervision Research Study: Workbook**  Please write your answers overleaf. These could be in bullet point form or as brief statements. We are looking for something like a concise paragraph on each, rather than long, detailed answers.   \| **Name:** \|  \| \| --- \| --- \| \| **ID Number:** \|  \|   This research has been funded by the NIHR School for Social Care Research. This phase of the research has been approved by Kingston University Business Faculty Research Ethics Committee (FR 16 09) and is supported by the Association of Directors of Adult Social Services (RG15 024).  **Case Study CI**  **Problem 1**  **Recommended decision 1**  **Problem 2**  **Recommended decision 2**  **Problem 3**  **Recommended decision 3**  **Problem 4**  **Recommended decision 4**  **Problem 5**  **Recommended decision 5**  **You have also been given a list of Key Principles Underpinning Decision Making in Adult Social Care. Please identify by Principle Number the five which most reflect the principles you had in mind in arriving at the specific recommendations you made about CI.**   \| Principle No \| Principle No \| Principle No \| Principle No \| Principle No \| \| --- \| --- \| --- \| --- \| --- \| \|  \|  \|  \|  \|  \|   **Case Study EK**  **Problem 1**  **Recommended decision 1**  **Problem 2**  **Recommended decision 2**  **Problem 3**  **Recommended decision 3**  **Problem 4**  **Recommended decision 4**  **Problem 5**  **Recommended decision 5**  **You have also been given a list of Key Principles Underpinning Decision Making in Adult Social Care. Please identify by Principle Number the five which most reflect the principles you had in mind in arriving at the specific recommendations you made about EK.**   \| Principle No \| Principle No \| Principle No \| Principle No \| Principle No \| \| --- \| --- \| --- \| --- \| --- \| \|  \|  \|  \|  \|  \|   **We now need to know how you found completing these tasks and how you felt about them. Please indicate on the scales below how strongly you agree or disagree with each statement.** (For A-E, please find definitions below)   1. **Mental Demand:** How much mental and perceptual activity was required?  \| Low \| 1 \| 2 \| 3 \| 4 \| 5 \| 6 \| 7 \| High \| \| --- \| --- \| --- \| --- \| --- \| --- \| --- \| --- \| --- \|  1. **Temporal Demand:** How much time pressure did you feel?  \| Low \| 1 \| 2 \| 3 \| 4 \| 5 \| 6 \| 7 \| High \| \| --- \| --- \| --- \| --- \| --- \| --- \| --- \| --- \| --- \|  1. **Performance:** How successful do you think you were?  \| Poor \| 1 \| 2 \| 3 \| 4 \| 5 \| 6 \| 7 \| Good \| \| --- \| --- \| --- \| --- \| --- \| --- \| --- \| --- \| --- \|  1. **Effort:** How hard did you have to work?  \| Low \| 1 \| 2 \| 3 \| 4 \| 5 \| 6 \| 7 \| High \| \| --- \| --- \| --- \| --- \| --- \| --- \| --- \| --- \| --- \|  1. **Frustration:** How insecure did you feel during the task?  \| Low \| 1 \| 2 \| 3 \| 4 \| 5 \| 6 \| 7 \| High \| \| --- \| --- \| --- \| --- \| --- \| --- \| --- \| --- \| --- \|   **Rating Scale Definitions**  **Mental Demand:** How much mental and perceptual activity was required (e.g. thinking, deciding, calculating, remembering, looking, searching etc.)? Was the task easy or demanding, simple or complex, exacting or forgiving?  **Temporal Demand:** How much time pressure did you feel due to the rate or pace at which the tasks or task elements occurred? Was the pace slow and leisurely or rapid and frantic?  **Performance:** How successful do you think you were in accomplishing the goals of the task set by the researcher (or yourself)? How satisfied were you with your performance in accomplishing these goals?  **Effort:** How hard did you have to work (mentally) to accomplish your level of performance?  **Frustration:** How insecure, discouraged, irritated, stressed and annoyed versus secure, gratified, content, relaxed and complacent did you feel during the task?   1. To what extent would you feel able to make decisions about these cases yourself rather than make recommendations to a supervisor?  \| Completely Would Not \| 1 \| 2 \| 3 \| 4 \| 5 \| 6 \| 7 \| Completely Would \| \| --- \| --- \| --- \| --- \| --- \| --- \| --- \| --- \| --- \|  1. To what extent did your sense of authority as a social worker affect your decisions or recommendations on these cases?  \| Completely Did Not \| 1 \| 2 \| 3 \| 4 \| 5 \| 6 \| 7 \| Completely Did \| \| --- \| --- \| --- \| --- \| --- \| --- \| --- \| --- \| --- \|  1. To what extent did you feel accountable for your decisions or recommendations on these cases?  \| Completely Did Not \| 1 \| 2 \| 3 \| 4 \| 5 \| 6 \| 7 \| Completely Did \| \| --- \| --- \| --- \| --- \| --- \| --- \| --- \| --- \| --- \|  1. To what extent did feeling that you had to account for your decisions or recommendations on these cases affect what you decided or recommended?  \| Not At All \| 1 \| 2 \| 3 \| 4 \| 5 \| 6 \| 7 \| Completely \| \| --- \| --- \| --- \| --- \| --- \| --- \| --- \| --- \| --- \|  1. To what extent would you feel responsible for the outcome from your decisions or recommendations on these cases?  \| Not At All \| 1 \| 2 \| 3 \| 4 \| 5 \| 6 \| 7 \| Completely \| \| --- \| --- \| --- \| --- \| --- \| --- \| --- \| --- \| --- \|  1. To what extent would you feel regret if your decisions or recommendations on these cases led to a poor outcome for the client?  \| Not At All \| 1 \| 2 \| 3 \| 4 \| 5 \| 6 \| 7 \| Completely \| \| --- \| --- \| --- \| --- \| --- \| --- \| --- \| --- \| --- \|  1. These are the sort of cases I would normally be asked to make decisions or recommendations on.  \| Strongly Disagree \| 1 \| 2 \| 3 \| 4 \| 5 \| 6 \| 7 \| Strongly Agree \| \| --- \| --- \| --- \| --- \| --- \| --- \| --- \| --- \| --- \|  1. The vignettes were easy to read and understand.  \| Not At All \| 1 \| 2 \| 3 \| 4 \| 5 \| 6 \| 7 \| Completely \| \| --- \| --- \| --- \| --- \| --- \| --- \| --- \| --- \| --- \|  1. The task instructions were easy to read and understand.  \| Not At All \| 1 \| 2 \| 3 \| 4 \| 5 \| 6 \| 7 \| Completely \| \| --- \| --- \| --- \| --- \| --- \| --- \| --- \| --- \| --- \|  1. How many years is it since you qualified as a social worker?   **Thank you for your participation in this research. Your support is greatly appreciated.** |
| --- | --- | --- | --- | --- | --- | --- | --- | --- | --- | --- | --- | --- | --- | --- | --- | --- | --- | --- | --- | --- | --- | --- | --- | --- | --- | --- | --- | --- | --- | --- | --- | --- | --- | --- | --- | --- | --- | --- | --- | --- | --- | --- | --- | --- | --- | --- | --- | --- | --- | --- | --- | --- | --- | --- | --- | --- | --- | --- | --- | --- | --- | --- | --- | --- | --- | --- | --- | --- | --- | --- | --- | --- | --- | --- | --- | --- | --- | --- | --- | --- | --- | --- | --- | --- | --- | --- | --- | --- | --- | --- | --- | --- | --- | --- | --- | --- | --- | --- | --- | --- | --- | --- | --- | --- | --- | --- | --- | --- | --- | --- | --- | --- | --- | --- | --- | --- | --- | --- | --- | --- | --- | --- | --- | --- | --- | --- | --- | --- | --- | --- | --- | --- | --- | --- | --- | --- | --- | --- | --- | --- | --- | --- | --- | --- | --- | --- | --- | --- | --- | --- |
