# Supplementary material for: Development of a methodology for measuring the quality of statutory social workers’ complex decision-making
Source: PLoS One. 2025 Jun 20;20(6):e0325432. doi: 10.1371/journal.pone.0325432 (PMC12180715; doi:10.1371/journal.pone.0325432)
Supplement: S10 — (DOCX) [file pone.0325432.s010.docx]

**S8.** **Case Vignette Scoring Templates**

**Scoring Template AG**

| **Appropriate Judgements** |
| --- |
| It is not clear whether the provision of social work has been included in either the care and support plan or the support plan. |
| AG cannot enter into a financial contract |
| AG might be subject to undue influence from her friend |
| Possible financial abuse by daughter |
| Daughter’s possible withdrawal of care |
| AG is making a decision in connection with her care which she may not have the capacity to make |
| AG is making a decision to continue with a friendship, the consequences of which she may not have the mental capacity to understand or she may, alternatively, be making an unwise decision. |
| AG’s daughter is proposing to take steps which could have adverse consequences on family relationships |
| It is alleged that AG’s dementia might be worsened by her friendship |
| AG may not have the mental capacity to weigh any benefits of continuing the friendship against the disbenefits of possible worsened dementia |
| **Appropriate Decisions** |
| Ascertain whether the social work client is AG or the carer. If it is not clear from the care and support plan or the support plan, assess accordingly. |
| Ensure that AG does not enter into a contract |
| Investigate whether or not AG is being subject to abuse by the lodger |
| Establish whether there is sufficient cause to suspect abuse or neglect (because of the daughter threatening to withhold the cash) |
| Carers assessment if only withdrawal of care identified |
| Establish whether or not AG has the mental capacity to decide on the implications of losing her daughter as a carer if she continues with the relationship with her friend |
| Assess AG’s mental capacity to understand the implications of her friendship for her family relationships |
| Provide or arrange counselling or other types of social work or other help to assist resolve the family relationship problem |
| Ascertain whether AG’s dementia might be worsened by this friendship |
| If so, assess whether she has the mental capacity to understand this. |

**Scoring Template BH**

| **Appropriate Judgements** |
| --- |
| Apparently persistent behaviour has led Housing to ask for social work help |
| It is not clear whether the provision of social work is already included in a care and support plan for BH |
| Capacitous refusal to comply |
| BH might present a risk of abuse to the neighbours’ children because of his behaviour |
| There is a possibility of abuse of an adult in need of care and support by his neighbours. |
| There is an imbalance between BH’s right to tolerance and his neighbours’ rights to have him meet his tenancy agreement in respect of them and this imbalance is adversely affecting his social well-being |
| BH’s daily life might be undignified |
| Current social care services may be insufficient |
| BH is refusing the change of services on offer because he feels they would be too restrictive for him |
| Conflict with neighbours might have adverse effect on mental state |
| Cooperate with Housing in their statutory duty |
| If social work is not a service included within a current care and support plan, then assess the needs for social work to be provided as all or part of a care and support plan |
| **Appropriate Decisions** |
| Assess whether there is a reasonable prospect of social work complying |
| Assess whether BH’s capacitous refusal to observe reasonable requirements is persistent and unequivocal |
| Assess whether other measures such as eviction or anti-social behaviour measures may be more appropriate |
| Assess whether BH is a risk to local children |
| Assess whether BH is unable to protect himself against abuse as a result of his needs for care and support |
| To assess what would be an appropriate balance between BH and his neighbours |
| Provide counselling or other types of social work to help achieve it (an appropriate balance). |
| Assess whether or not BH is the best judge of his own well-being in this matter (lack of dignity) |
| To provide counselling or other types of social work to address his possible undignified manner of living |
| To reassess the care services needed |
| To assess impact of conflict on mental state |

**Scoring Template CI**

| **Appropriate Judgements** |
| --- |
| A review of CI’s care and support is needed because of the deterioration in her physical well-being |
| CI is experiencing a life changing illness and it is possible that adjustment is emotionally difficult for her |
| There is reason to believe that CI is experiencing or is at risk of abuse |
| There might be incidents of abuse observed by the independent personal assistant which are not being reported to the council and it may be that the PA cannot be relied on to do this |
| CI might be unable to protect herself from abuse because of her needs for care and support and, if so, there is an absolute duty to cause enquiries to be made |
| CI’s wishes do not seem to be in her best interests in that they would not protect her from the possible abuse |
| There might be incidents of abuse observed by the independent personal assistant but unknown to the council and the PA might not know what is expected of them |
| The loss of CI’s roles as a paid worker and as the manager of the family household appear to be a significant loss to her and could possibly exacerbate a deterioration in her functioning and also increase family tension at home |
| Information which might be essential to CI’s well-being has been given in confidence. If safeguarding enquiries are initiated, then the confidences cannot be respected and this could have an adverse effect on family and personal relationships. |
| The changing roles within the family appear to be causing difficulties for CI’s husband with possible adverse consequences for CI’s well-being |
| The daughter is a carer of her mother and is looking tired and anxious, which suggests that she may be finding the caring difficult |
| No issue because there is no reason to believe that CI’s husband either provides or intends to provide care to CI. |
| **Appropriate Decisions** |
| To assess whether the amount of care is sufficient to meet CI's needs, in the light of the apparent recent deterioration |
| To assess how well CI is adjusting emotionally and whether she would benefit from counselling or other type of social work and would wish to receive it |
| Assess CI’s needs for counselling or other types of social work concerning the possible abuse |
| Assess whether direct payments are the best means of promoting CI’s well-being so far as relating to protection from abuse |
| Interview CI to ascertain if there is reasonable cause to suspect that she is unable to protect herself because of her needs for care and support |
| Decide, on the facts already available, whether she is experiencing or is at risk of abuse. If we conclude that she is, then we cannot accept her wishes as a reason for not continuing with an assessment of how to promote her well-being in this regard |
| To ensure that the PA is receiving sufficient information on safeguarding to make direct payments an appropriate service and to promote CI’s control over her day-to-day life |
| To discuss with CI the possibility of day services, of which she might be unaware |
| To assess whether CI wishes to and is working with her PA in such a way as to maximise her control over the management of the household |
| If safeguarding enquiries are initiated, to provide counselling or other type of social work with the family to minimise the impact of the disclosures. |
| To offer counselling or other type of social work either to him alone or to them as a couple |
| To establish with the daughter whether she wishes to have a carer’s assessment |
| Do nothing because there is no reason to believe that CI’s husband either provides or intends to provide care to CI. |

**Scoring Template EK**

| **Appropriate Judgements** |
| --- |
| EK is engaging in acts of violence which, if they are beyond her control, compromise her dignity |
| Her physical well-being may be at risk because she is walking out of Headway and it might be unsafe for her to do so |
| Her physical well-being may be at risk because she might walk out of her flat in the future and it might be unsafe for her to do so |
| Her emotional well-being needs promoting because she is demonstrating frustration at Headway for unknown reasons |
| She wants to continue attending Headway but, because she is walking out and potentially placing herself at risk, that decision might be beyond her control |
| She wants to remain living in her flat but this decision might ultimately be outside her control should she be found to be at risk from walking out of her flat |
| The benefits at Headway seem to have reached a plateau but she might lose the benefits of what she has gained were she to stop attending because of this |
| Her social well-being is compromised by her acts of violence and the placement is at risk |
| The parents have expressed a clear proposal which must be taken into account but might not be followed and, if it is not, they might find this hard to accept |
| Her flat might not be suitable for day activities should she leave Headway |
| The consultant neurologist and/or EK’s parents have suggested that EK needs residential care and their reasons must be taken into consideration |
| It is possible that a firm difference of opinion might emerge between EK and her parents which might render it inappropriate for them to represent and support her in the review and it is possible that none of her other supportive friends or family would be appropriate either |
| **Appropriate Decisions** |
| Specific assessment of why she is being violent at Headway |
| Specific assessment of how unsafe it is for her to walk out of Headway unaccompanied; |
| Specific assessment of how likely it is that she will walk out of her flat; how unsafe it would be for her to do so |
| Specific assessment to arrive at an understanding of the level and nature of her frustration...with a view to recommissioning the programme at Headway |
| To determine whether she is the best judge of her own well-being in her decisions to walk out and whether she has the mental capacity to decide to walk out of Headway. |
| To determine whether she would be the best judge of her own well-being should she decide to walk out and put herself at risk and whether she has the mental capacity to decide to walk out of her flat |
| To assess if there is another day provision which can lead to more improvement |
| Specific assessment of the balance of EK’s needs for day services set against the needs of Headway staff not to be attacked |
| Counselling or other types of social work with parents to help reconcile differences of opinion |
| Specific assessment of the suitability of her accommodation for day activities, involving an OT |
| To interview the consultant neurologist and/or EK’s parents to assess their reasons for believing that EK needs residential care |
| To assess whether any of EK’s friends or family are appropriate to represent and support her in the review and, if not, to arrange for an independent advocate |
