# Supplementary material for: Development of a methodology for measuring the quality of statutory social workers’ complex decision-making
Source: PLoS One. 2025 Jun 20;20(6):e0325432. doi: 10.1371/journal.pone.0325432 (PMC12180715; doi:10.1371/journal.pone.0325432)
Supplement: Attachment — Submitted filename: Response to Reviewers.docx [file pone.0325432.s012.docx]

**Response to Reviewers**

| 1. | Please ensure that your manuscript meets PLOS ONE's style requirements, including those for file naming. | *We have reviewed the manuscript to ensure that it meets PLOS One style requirements. We have made some minor revisions to the main body and author formatting to ensure compliance – thank you for the guidance on this.* |
| --- | --- | --- |
| 2. | Please state what role the funders took in the study. If the funders had no role, please state: "The funders had no role in study design, data collection and analysis, decision to publish, or preparation of the manuscript." If this statement is not correct you must amend it as needed. Please include this amended Role of Funder statement in your cover letter; we will change the online submission form on your behalf. | *We have amended the Role of Funder statement as follows (lines 38-43).*  *Funding. This project, including contributions for authors ^1^ and ^3^, was funded by the School for Social Care Research, a national research school established by the National Institute of Health and Care Research. The funders had no role in study design, data collection and analysis, decision to publish, or preparation of the manuscript. The views expressed are those of the authors and not necessarily those of the National Institute of Health and Care Research or the Department of Health and Social Care.*  *Thank you for changing the online submission form.* |
| 3. | Please review your reference list to ensure that it is complete and correct. If you have cited papers that have been retracted, please include the rationale for doing so in the manuscript text, or remove these references and replace them with relevant current references. Any changes to the reference list should be mentioned in the rebuttal letter that accompanies your revised manuscript. If you need to cite a retracted article, indicate the article’s retracted status in the References list and also include a citation and full reference for the retraction notice. | *We have reviewed the reference list and believe it to be complete and correct, with no retracted papers cited.* |
| 4. | Reviewer One Comment One  The research sets out to develop a measure of decision-making quality. I would like to see some brief comment added to acknowledge that that this measure was based solely on legal principles. The research aims indicate that the study was to develop a methodology to measure quality of decision-making processes 'including' application of legal principles. While these legal principles may incorporate or overlap with other factors in quality (such as taking into account service users' views and preferences) they appear to be the sole basis of the measure, not one which was 'included' in a wider methodology. | *We are grateful for this feedback and have addressed this throughout the manuscript. For example, we have revised the Abstract to include the statement that: “Central to our methodology was social workers’ consideration of key legal principles.” (lines 61-62)*  *We have revised the Introduction to include the statement that: “Our objectives were to develop a methodology to (1) measure the quality of decision-making processes, (2) insofar as legal principles relevant to local authority adult social care appeared to have been considered in decision-making, and (3) explore underpinning cognitive processes.” (lines 144-145)*  *We have revised the Discussion to include the statement that: “Collaborating with expert professionals, we developed a new methodology to measure the quality of social workers’ decision-making, insofar as key legal principles for the adult social care setting are considered, and investigate underpinning cognitive processes.” (lines 653-654)* |
| 5. | Reviewer One Comment Two  References to legislation with nationalities in brackets were not explained and could be confusing. E.g. Care Act 2014 (England) or Human Rights Act 1988 (England) appear to be indicating the geographical extent of this legislation but it is not clear. This could be amended or explained to strengthen this minor element of presentation. | *We have addressed this by stating which is the relevant jurisdiction for each Act when first mentioned and thereafter removing the jurisdiction reference.* |
| 6. | Reviewer Two Comment One  I would recommend that the authors state how they are defining complex decision making in the introduction. In lines 88-90, the authors state that, “The profession has defined complex decision-making as arising when an adult’s judgement about their well-being or their wishes about how to promote it put their well-being at risk of harm….”. However, this example is problematic because it reflects one form of complex decision-making in social work, where a range of other complex decisions exist. It is ok to use reference 6 as an example of an important type of complex decision-making, but the authors need to give a clearer definition of this concept, drawing on decision-making literature. A lot of this information is presented in the methods section (lines 163-177), so I would recommend this be sketched out in the introduction with pointers to the methods section. | *We agree and have revised the Introduction to reflect that this is one type of complex decision-making by including the statement that: “In adult social care, one important type of complex decision-making is the decision-making required when an adult’s judgement about their well-being or their wishes about how to promote it put their well-being at risk.”* (originally lines 88-90, now lines 100-102).  *We have summarised decision-making stages, drawing on decision-making literature, in the Introduction and referred to our expansion on this in our Method section* (lines 92-96). |
| 7. | Reviewer Two Comment Two  When reading the introduction, I thought that the paper was focussing specifically on adult safeguarding as the examples given in the introduction all pertain to this. You give safeguarding as an example in lines 88-90 and go onto give examples of self-neglect (also categorised as safeguarding under the Care and Support Statutory guidance) and findings from Safeguarding Adults Reviews (lines 106-122). However, it becomes clear later in the paper that the remit for the paper is broader than this focussing on principles of public body decision making / legal principles. Given this, I think your examples given in the introduction should be broader. This problem recurs in the examples given also pertain to safeguarding whilst the focus of the project is broader. I would also suggest this section is amended, so the scope of the project is better represented (lines 643-644). | *We are grateful for this advice and have revised the Introduction by considerably reducing the narrative on safeguarding adults reviews and making clear that, whilst complex decision-making may arise in safeguarding situations, complex decision-making is not at all limited to safeguarding. We have referred to ways in which safeguarding adults reviews might shed relevant light on the quality of complex decision-making, such as better balancing of legal duties, but have moved away from a detailed description of safeguarding adults reviews. We have then referred, as per the original manuscript, to the pressing need for a methodology to measure the quality of decision-making (*now lines 113-132).  *We have also referred to “complex decision-making which may arise when an adult’s judgement about their well-being or wishes about how to promote it may, in the circumstances, put their well-being at risk.”, removing the word harm here and in one or two other places, to emphasise that complex decision-making is broader than safeguarding (*line 650). |
